# Comparison of the accuracy of two techniques for three-dimensional digital indirect bonding of orthodontic brackets: A randomized controlled trial

**DOI:** 10.1590/2177-6709.29.3.e2423117.oar

**Published:** 2024-07-08

**Authors:** Ahmed Abdelhalim MAHRAN, Wael Mubarak REFAI, Ahmed Shawky HASHEM

**Affiliations:** 1 Beni-Suef University, Faculty of Dentistry, Department of Orthodontics (Beni Suef, Egypt).; 2 Minia University, Faculty of Dentistry, Department of Orthodontics (Minia, Egypt).

**Keywords:** Indirect bracket bonding, Transfer tray, Transfer error, 3D printing, Colagem indireta de braquetes, Guia de transferência, Erro de transferência, Impressão 3D

## Abstract

**Objective:**

This study aimed to clinically compare the accuracy of bracket positioning between three-dimensionally (3D) printed indirect bonding trays and vacuum-formed trays made over 3D-printed models.

**Material and Methods:**

Fourteen patients, planned for fixed orthodontic therapy, were randomly divided into two equal groups. For both groups, both dental arches were scanned, to acquire virtual models, brackets were virtually positioned from central incisors to second premolars, and scans for the final bracket positions were performed. In the first group, transfer trays were 3D-printed. In the second group, virtual models were 3D-printed, and vacuum-formed soft sheets were thermoformed on the printed model. Teeth were indirectly bonded and then scanned. Superimposition of the virtual and the final bracket positioning scans was performed to measure linear and angular deviations in brackets positions.

**Results:**

The first group showed significantly less occlusogingival and buccolingual linear errors than the second group. No significant differences in angular deviations were found between both groups. The frequencies of clinically acceptable linear errors within 0.5 mm and angular errors within 2° showed no statistically significant difference between both groups (*p*> 0.05 for all measurements). The transfer errors in both groups showed linear directional biases toward the mesial, gingival and labial directions. There was no statistically significant difference in the rate of immediate debonding between both groups (10.7% and 7.1% for the first and the second groups, respectively, *p*=0.295).

**Conclusions:**

3D-printed indirect bonding trays were more accurate than vacuum-formed trays, in terms of linear deviations. Both types of trays showed similar angular control.

## RESUMO

**Objetivo:**

Esse estudo teve como objetivo comparar clinicamente a precisão no posicionamento de braquetes usando guias de colagem indireta impressas tridimensionalmente (3D) e guias termoformadas a vácuo em cima de modelos impressos em 3D.

**Material e Métodos:**

Quatorze pacientes, planejados para tratamento ortodôntico fixo, foram divididos aleatoriamente em dois grupos de igual tamanho. Em ambos os grupos, as duas arcadas dentárias foram escaneadas para a criação dos modelos virtuais, os braquetes foram posicionados virtualmente dos incisivos centrais aos segundos pré-molares, e foram feitos os escaneamentos das posições finais dos braquetes. No primeiro grupo, as guias de transferência foram impressas em 3D. No segundo grupo, os modelos virtuais foram impressos em 3D e as guias foram termoformadas a vácuo em cima dos modelos impressos. Os braquetes foram colados de forma indireta e depois feitos os escaneamentos. Uma sobreposição das posições planejadas virtualmente e do escaneamento com os braquetes na posição final foi realizada para medir os desvios lineares e angulares nas posições dos braquetes.

**Resultados:**

O primeiro grupo apresentou significativamente menos erros lineares oclusogengivais e vestibulolinguais do que o segundo grupo. Não foram encontradas diferenças significativas nos desvios angulares entre os dois grupos. As frequências de erros lineares clinicamente aceitáveis menores que 0,5 mm e de erros angulares menores que 2° não apresentaram diferença estatisticamente significativa entre os dois grupos (*p* > 0,05 para todas as medições). Os erros de transferência em ambos os grupos mostraram tendências a desvios lineares nas direções mesial, gengival e labial. Não houve diferença estatisticamente significativa entre os dois grupos para a taxa de descolagem imediata (10,7% e 7,1% para o primeiro e o segundo grupos, respectivamente, *p*=0,295).

**Conclusões:**

As guias de colagem indireta impressas em 3D foram mais precisas em termos de desvios lineares do que as guias termoformadas a vácuo. Os dois tipos de guias apresentaram controle angular semelhante.

## INTRODUCTION

Andrews, in 1979, presented the first fully preset appliance, the Straight-wire appliance.^1^ It consisted of specially designed brackets with prescriptions for tip, in and out, and torque specific for each tooth.^1^ ^,^ ^2^ The positioning of the preset brackets on the tooth crown regulates the tooth’s final tip, torque, height and rotation.^3^ Appropriately positioned brackets ensure properly aligned crowns and roots, and diminish the need for additional archwire adjustments, resulting in decrease in treatment duration and appropriate final occlusion.^4^ Inadequate bracket positioning can make the best customized prescription inefficient.^3^ ^,^ ^4^

Indirect bonding (IDB) was originally presented by Silverman et al.^5^, in 1972, as a procedure comprising transfer of orthodontic brackets from working models to the patient’s dentition, using transfer trays. This was a trial to progress to bandless orthodontic treatments, with the advantage of increasing the possibilities of managing borderline cases with a nonextraction approach.^5^ The routine indirect bonding procedure embraces bonding of brackets to plaster models and subsequently transferring them to the patient’s mouth, by means of an indirect bonding transfer tray made of silicon or thermoplastic material.^6^ ^-^ ^8^

Although IDB does not achieve perfect bracket positioning, IDB trays produce greater bracket positioning accuracy than the frequently used direct bonding procedures.^6^ ^,^ ^9^ This higher precision is a consequence of placement of the brackets in absence of clinical conditions and variables that can impair the direct technique, as moisture contamination, patient cooperation or rushed appointments.^10^

Recently, software development has enabled digital planning for bracket placement. Digital scanning is used either for the physical models, with a desktop scanner, or for the patient’s mouth, using an intraoral scanner.^11^ As the brackets are positioned on the models, a transfer tray including the brackets in their proposed positions is constructed or printed, and placed inside the patient’s mouth, to initiate bonding.^11^ ^,^ ^12^

Most of the studies assessing the accuracy of indirect bonding were *in-vitro* studies, utilizing models to assess indirect bonding accuracy. Schmid et al.^13^ appraised the transfer accuracy of silicone and double vacuum-formed trays, and suggested that both displayed excellent precision, particularly silicone trays. Niu et al.^14^ compared the accuracy of two transfer trays on dental models, and concluded that three-dimensionally (3D) printed trays are more precise than vacuum-formed trays, especially in the horizontal bracket control. However, these *in-vitro* studies did not cogitate the impact of ease of bonding by the operator and moisture control.

Although previous studies^13^ ^,^ ^14^ assessed the reliability of different methods for indirect orthodontic brackets bonding, recent studies including systematic reviews and meta-analyses^15^ ^-^ ^17^ recommended additional *in vivo* randomized controlled trials to assess the accuracy of indirect bonding techniques and to report the validity of the adopted approaches, considering the evaluation of their accuracy. Therefore, the present study aimed to clinically compare the accuracy of indirect bracket bonding using 3D-printed trays or vacuum-formed trays made over 3D-printed models. The accuracy was assessed by measuring the difference between the desired and the final bracket positions, subsequent to the indirect bonding. This study was proposed to evaluate the null hypothesis that the precision of bracket positioning would not be influenced whether indirect bonding was executed using 3D-printed transfer trays or vacuum-formed trays.

## MATERIAL AND METHODS

This study was approved by the ethical committee of the Faculty of Dentistry, Minia University, Egypt (approval no. 369-2019). The sample size was defined based on a previous study^13^ comparing silicone and double vacuum-formed trays, by taking the means and standard deviations of the linear vertical transfer errors in comparison between both groups as a primary outcome. Considering 80% power, 0.05 level of significance, and 0.105 ± 0.078 mm and 0.071 ± 0.052 mm means ± standard deviations of the transfer errors in the first and the second groups, respectively, the minimum sample size needed to reveal statistically significant difference was 180 (90 per group). 

This study was designed as a randomized controlled trial, carried out on fourteen patients planned to wear upper and lower fixed orthodontic appliances. The study procedures were fully explained to all patients, and written consents were signed by them. The criteria for inclusion comprised full permanent dentition, good oral hygiene and less than 5 mm of crowding. The exclusion criteria were: enamel hypocalcification, bulky restorations involving the facial surfaces of teeth, or lost permanent teeth. A total of 28 dental arches with 280 teeth (all maxillary or mandibular incisors, canines or premolars) were included in this study. 

**Figure 1:**
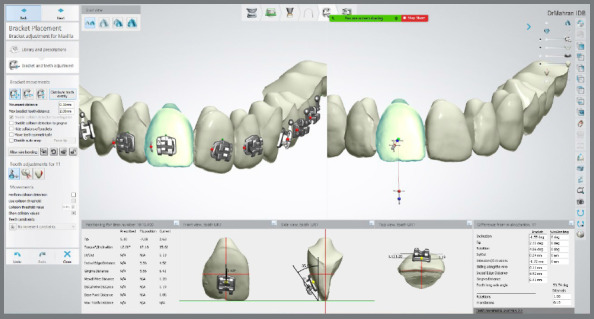
Virtual bracket positioning, from central incisors to second premolars.

Patients were randomly divided into two groups, by a clinician not participating in this study, using properly sealed envelopes. The means and standard deviations of the ages were 19.46± 2.18 and 20.52± 2.97 years for the first and the second groups, respectively. For both groups, maxillary and mandibular dental arches were scanned using an intraoral scanner (CS 3700, Carestream Dental, Georgia, USA) to obtain stereolithographic (STL) files, which were transferred to the Ortho Analyzer software (3Shape, Copenhagen, Denmark) to create the virtual models. Brackets (Morelli Max 0.022-in Roth prescriptions, Morelli, Brazil) were virtually positioned, from central incisors to second premolars (Fig. 1), and additional STL files for the final positions of the brackets were created, as reference for subsequent superimpositions. 

**Figure 2:**
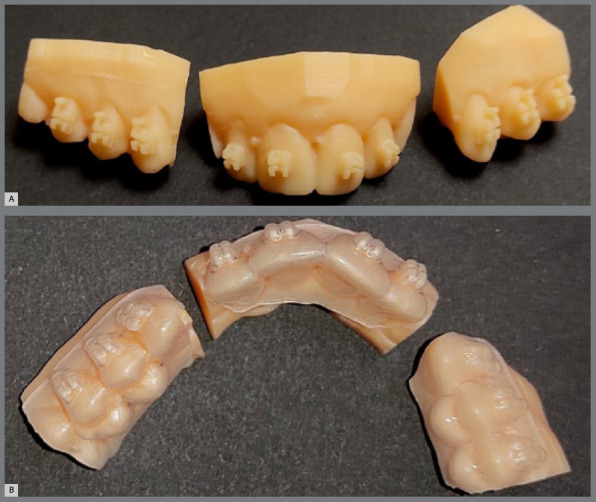
A) 3D-printed virtual models, in three segments. B) Transfer trays thermoformed on the 3D-printed model.

In the vacuum-formed tray group, hard Model 2.0 resin (Nextdent, Netherlands) was used in a Mogassam 3D printer (DentCase, Delaware, USA) to print the virtual models in three segments: one segment including the central and lateral incisors, and two segments involving canines and premolars on both sides. Subsequently, vacuum-formed soft sheets (Bioplast, Scheu, Germany) of 1-mm thickness were thermoformed on the printed model, using a pressure molding machine (Ministar, Scheu, Germany), to obtain the vacuum-formed transfer trays with negative replica of brackets. The transfer trays were designed with their contours extending immediately gingival to the brackets, either buccally or labially, and covering the entire palatal or lingual surfaces. The brackets were then fitted in the vacuum-formed transfer trays (Fig 2).

**Figure 3:**
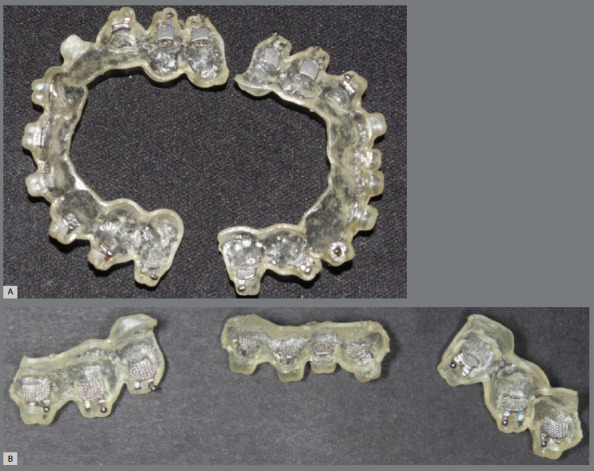
A) Transfer tray 3D-printed using biocompatible resin. B) 3D-printed transfer tray split in three segments, and loaded with the brackets.

In the 3D-printed trays group, STL files were transferred to 3Shape Appliance Designer software (3Shape, Copenhagen, Denmark). Transfer trays were subsequently 3D-printed with 1-mm thickness, using a Mogassam 3D printer (dentCase, Delaware, USA), with biocompatible flexible Ortho IBT resin (Nextdent, Netherlands), with the same proportions of the first group. Accordingly, they were split into the same three segments as the first group, and loaded with the brackets (Fig 3).

### INDIRECT BONDING PROCEDURE

All teeth from the right to the left second premolar were polished with brush and pumice (Prophy Paste, Belvedere, Kent, UK), etched with 37% phosphoric acid (Meta etchant, Meta Biomed, Korea) for twenty seconds, and washed for 30 seconds. The teeth were properly dried with air jets and isolation was carried out, paying attention to the lingual and palatal surfaces. 

Ortho Solo bonding agent (Ormco, California, USA) was applied to coat the etched surfaces. Light-cured adhesive resin (Greengloo, Ormco, California, USA) was applied on the meshes of the bases of the brackets in the transfer trays. The transfer trays, including the brackets adjusted to their positions, were consistently seated intraorally on the teeth, using light and constant finger pressure parallel to the occlusal plane, by the operator’s right hand. The clear tray design permitted visual confirmation of the appropriate tray position throughout the light-curing procedure. Twenty seconds of light-curing (Woodpecker, China) were used on each side (gingival, labial and incisal) for each bracket. The tray was then removed and the excess of bonding agent surrounding the brackets was removed.

Subsequently, the teeth with the bonded brackets were scanned using an intraoral scanner (CS 3700, Carestream Dental, Georgia, USA), and the STL files of the post-bonding models were kept for subsequent superimpositions. 

### SUPERIMPOSITIONS

**Figure 4:**
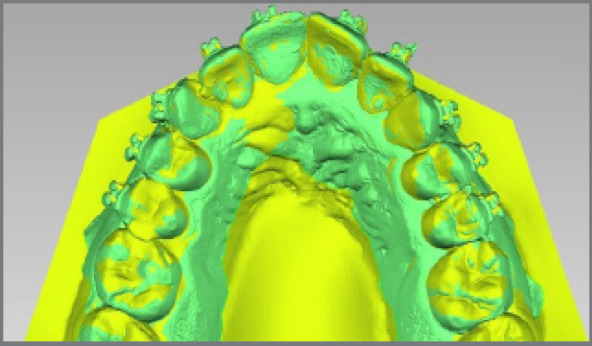
Superimposition of the model with the virtually positioned brackets (Green color) and the model with the bonded brackets (Yellow color).

Superimpositions of the first model (with the virtually positioned brackets) and the second model (with the bonded brackets) were performed using Geomagic Qualify software v. 12.0 (3D systems, North Carolina, USA), with a best-fit algorithm (Fig 4). A linear coordinate system (X, Y and Z axes) was made for all brackets, taking into consideration the midpoint of each bracket base. 

**Figure 5:**
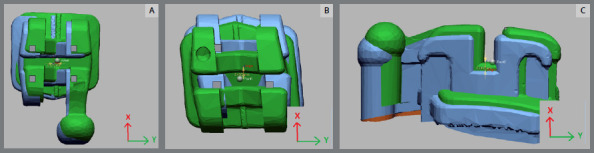
Measurements of the linear discrepancies between the virtually positioned and the intraorally bonded brackets: A) in the mesiodistal direction, B) in the occlusogingival direction, C) in the buccolingual direction.

**Figure 6:**
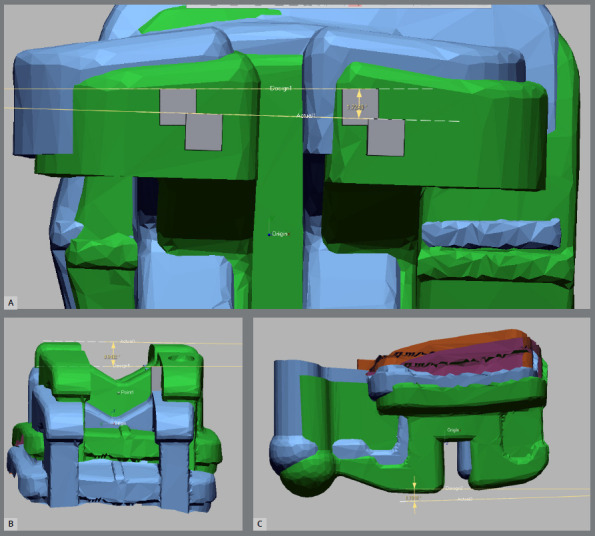
Measurements of the angular discrepancies between the virtually positioned and the intraorally bonded brackets: A) mesiodistal tip, B) rotation, C) buccolingual torque.

The center of the brackets in both models was defined, and mesiodistal (MD), occlusogingival (OG) and buccolingual (BL) linear deviations were determined as the linear distances between the center of the brackets in both models (Fig 5). Regarding angular deviations (tipping, rotation and torquing), vertical and horizontal lines extending between the edges of the bracket wings were drawn, and superimpositions between both scans, incorporating vertical and horizontal lines, were performed. Angles between the superimposed vertical and horizontal lines were measured, to determine the angular deviations (Fig 6). Threshold limits for linear and angular transfer accuracies in this study were 0.5 mm and 2°, respectively.

The number of brackets that failed to bond during transfer tray removal was recorded for both groups.

### STATISTICAL ANALYSIS

After one week of the measurements, the same operator re-measured both the linear and angular transfer errors in three patients for each group. Interclass correlation coefficient (ICC) was used to evaluate reliability of the measurements. 

The means and standard deviations of all variables comprised in this study were calculated. All variables were investigated for normality using Kolmogorov-Smirnov test, resulting in *p*˃ 0.05 for all of them, revealing normal distribution. The level of significance was established at *p* ≤ 0.05. Statistical analysis was accomplished using Statistical Package for Social Sciences v. 20 software (SPSS, Chicago, USA).

One sample *t*-test was used to clarify whether the mean transfer errors were statistically significant within the designated acceptable limits of 0.5 mm for linear measurements and 2 degrees for angular measurements. Bracket transfer was considered accurate for *p* ≤ 0.05 and for transfer deviation mean less than the preset limits. Independent samples *t*-test was used to compare the mean transfer errors between both groups. Comparison of the transfer errors between different teeth categories (incisors, canines and premolars) in both groups was analyzed by one-way analysis of variance (ANOVA). Qui-square test was used to compare the frequencies of clinically acceptable errors, directional bias and bracket debonding between both groups. 

## RESULTS

ICC was greater than 0.963, with *p*<0.001 for all measurements, demonstrating excellent method reliability and agreement between the different readings. There was no statistically significant difference in the mean ages between both groups (*p*= 0.141). The amounts of crowding in the upper arch were 3.59 ± 0.84 mm and 3.07 ± 0.79 mm for the first and the second groups, respectively. The Little’s irregularity indices in the lower arch were 2.61 ± 0.35 mm and 2.38 ± 0.26 mm for the first and the second groups, respectively. There were no statistically significant differences in the means of the amounts of crowding and the Little’s irregularity indices between both groups (*p* = 0.136 and *p* = 0.462 for the first and the second groups, respectively).

All one-sided *t*-tests in both groups revealed acceptable transitional errors (*p* < 0.001) for all linear and angular dimensions, except for the torque errors, which showed higher means than the preset limit of 2°, despite presenting *p* < 0.001 (Table 1). 

**Table 1:** Comparison between the mean transfer errors and the preset limits of 0.5 mm for linear errors and 2° for angular errors, in both groups.

	Vacuum-formed tray group				3D-printed tray group			
	Upper arch		Lower arch		Upper arch		Lower arch	
	Mean±SD	P-value	Mean±SD	P-value	Mean±SD	P-value	Mean±SD	P-value
Mesiodistal (mm)	0.21±0.06	<0.001*	0.25±0.07	<0.001*	0.21±0.04	<0.001*	0.21±0.03	<0.001*
Occlusogingival (mm)	0.32±0.1	<0.001*	0.32±0.1	<0.001*	0.23±0.05	<0.001*	0.25±0.05	<0.001*
Buccolingual (mm)	0.21±0.16	<0.001*	0.27±0.15	<0.001*	0.19±0.11	<0.001*	0.18±0.15	<0.001*
Tip (degrees)	1.63±0.51	<0.001*	1.58±0.5	<0.001*	1.46±0.46	<0.001*	1.51±0.38	<0.001*
Rotation (degrees)	1.19±0.71	<0.001*	1.39±0.22	<0.001*	1±0.47	<0.001*	1.15±0.19	<0.001*
Torque (degrees)	2.43±0.66	<0.001*	2.51±0.72	<0.001*	2.41±0.57	<0.001*	2.47±0.64	<0.001*

* Significant at *p*≤0.05 (One-sided *t*-test).

Comparing both groups, there were no statistically significant differences in the transfer errors between them, except for the occlusogingival linear deviation in both arches (*p*=0.021 and *p*=0.001 for the first and the second groups, respectively) and the buccolingual linear deviation in the lower arch (*p*=0.001), with the vacuum-formed tray group showing higher mean errors than the 3D-printed tray group (Table 2).

**Table 2:** Comparison of the mean linear and angular transfer errors between both groups.

	Upper arch			Lower arch		
	Vacuum-formed tray group (Mean±SD)	3D-printed tray Group (Mean±SD)	P-value	Vacuum-formed tray group (Mean±SD)	3D-printed tray group (Mean±SD)	P-value
Mesiodistal (mm)	0.22±0.06	0.21±0.04	0.681	0.25±0.07	0.21±0.03	0.127
Occlusogingival (mm)	0.32±0.1	0.23±0.05	0.021*	0.32±0.1	0.25±0.05	0.001*
Buccolingual (mm)	0.21±0.16	0.19±0.11	0.391	0.27±0.15	0.18±0.15	0.001*
Tip (degrees)	1.63±0.51	1.46±0.46	0.673	1.58±0.5	1.51±0.38	0.375
Rotation (degrees)	1.19±0.71	1±0.47	0.354	1.39±0.22	1.15±0.19	0.572
Torque (degrees)	2.43±0.66	2.41±0.57	0.892	2.51±0.72	2.47±0.64	0.798

* Significant at *p*≤0.05 (Independent samples *t*-test).

For the linear errors, both groups showed the highest frequency of clinically acceptable errors, within 0.5 mm in the mesiodistal direction. The torque errors showed the smallest frequency of angular clinically acceptable errors, within 2 degrees, which did not exceed 60%. No statistically significant frequency differences were found between both groups (Table 3).

**Table 3:** Comparison of the frequency of clinically acceptable errors between both groups.

	Upper arch			Lower arch		
	Vacuum-formed tray group (%)	3D-printed tray group (%)	P-value	Vacuum- formed tray group (%)	3D-printed tray group (%)	P-value
Mesiodistal (mm)	98.5%	100	0.863	98.4	100	0.817
Occlusogingival (mm)	93.9	98.5	0.171	95.2	100	0.276
Buccolingual (mm)	95.5	98.5	0.31	96.8	98.4	0.549
Tip (degrees)	77.3	86.4	0.176	79	84.1	0.462
Rotation (degrees)	83.3	90.9	0.194	80.1	87.3	0.31
Torque (degrees)	56.1	59.1	0.725	52.4	54.8	0.783

* Significant at *p*≤0.05 (Qui-square test).

The transfer errors in both groups showed linear directional biases toward the mesial, gingival and labial directions. Moreover, both groups revealed angular directional biases with mesial crown tip, mesial out rotation and labial crown torque. There were no significant differences in all linear and angular biases between both groups, with *p*˃0.05 for all of them (Table 4). 

**Table 4:** Comparison of the percentages of directional bias of the transferred brackets between both groups.

		Upper arch					Lower arch				
		Vacuum-formed tray group		3D-printed tray group		P-value	Vacuum-formed tray group		3D-printed tray group		P-value
		n	%	n	%		n	%	n	%	
Mesiodistal (mm)	+	43	68.3	45	67.2	0.894	40	64.5	39	61.9	0.762
	-	20	31.7	22	32.8		22	35.5	24	38.1	
Occlusogingival (mm)	+	47	74.6	45	67.2	0.351	51	82.3	42	66.7	0.073
	-	16	25.4	22	32.8		11	17.7	21	33.3	
Buccolingual (mm)	+	46	73	45	67.2	0.467	51	82.3	46	73	0.215
	-	17	27	22	32.8		11	17.7	17	27	
Tip (degrees)	+	50	79.4	49	73.1	0.405	51	82.3	43	68.3	0.7
	-	13	20.6	18	26.9		11	17.7	20	31.7	
Rotation (degrees)	+	41	65.1	45	62.7	0.802	37	59.7	40	63.5	0.661
	-	22	34.9	22	37.7		25	40.3	23	36.5	
Torque (degrees)	+	48	76.2	45	67.2	0.254	47	75.8	48	76.2	0.96
	-	15	23.8	22	32.8		15	24.2	15	23.8	

Positive (+) means mesial, gingival and labial linear deviations. It also means mesial crown tip, mesial out rotation and labial crown torque. Negative (-) means the opposite. *Significant at *p*≤0.05 (Qui-square test).

Both groups showed no statistically significant differences in the transitional errors between different teeth groups (incisors, canines and premolars) in both dental arches, with *p*˃0.05 for all of them (Tables 5 and 6). 

**Table 5:** Comparison of linear and angular transfer errors between incisors, canines and premolars in the vacuum-formed tray group.

	Upper arch				Lower arch			
	Incisors	Canines	Premolars	P-value	Incisors	Canines	Premolars	P-value
	Mean±SD	Mean±SD	Mean±SD		Mean±SD	Mean±SD	Mean±SD	
Mesiodistal (mm)	0.22±0.03	0.2±0.04	0.2±0.04	0.282	0.21±0.03	0.22±0.03	0.21±0.04	0.274
Occlusogingival (mm)	0.22±0.04	0.24±0.07	0.23±05	0.392	0.25±0.05	0.24±0.05	0.25±0.06	0.799
Buccolingual (mm)	0.21±0.09	0.17±0.12	0.21±0.11	0.214	0.21±0.15	0.19±0.09	0.16±0.11	0.126
Tip (degrees)	1.36±0.42	1.5±0.44	1.54±0.51	0.402	1.44±0.38	1.44±0.4	1.62±0.36	0.183
Rotation (degrees)	0.9±0.32	1.11±0.31	1.1±0.4	0.541	1.14±0.33	1.19±0.1	1.2±0.12	0.462
Torque (degrees)	2.5±0.52	2.48±0.57	2.32±0.63	0.491	2.43±0.63	2.48±0.61	2.62±0.68	0.579

* Significant at *p*≤0.05 (One-way ANOVA).

**Table 6:** Comparison of linear and angular transfer errors between incisors, canines and premolars in the 3D-printed tray group.

	Upper arch				Lower arch			
	Incisors	Canines	Premolars	P-value	Incisors	Canines	Premolars	P-value
	Mean±SD	Mean±SD	Mean±SD		Mean±SD	Mean±SD	Mean±SD	
Mesiodistal (mm)	0.21±0.06	0.23±0.06	0.2±0.07	0.431	0.24±0.06	0.24±0.06	0.27±0.09	0.44
Occlusogingival (mm)	0.32±0.08	0.32±0.12	0.32±0.12	0.978	0.3±0.09	0.32±0.12	0.35±0.1	0.473
Buccolingual (mm)	0.17±0.16	0.26±0.17	0.28±0.19	0.062	0.32±0.11	0.23±0.16	0.28±0.18	0.213
Tip (degrees)	1.55±0.44	1.61±0.49	1.73±0.6	0.596	1.51±0.42	1.55±0.49	1.69±0.59	0.372
Rotation (degrees)	1.23±0.69	1.1±0.36	1.32±0.42	0.471	1.33±0.24	1.31±0.12	1.46±0.36	0.183
Torque (degrees)	2.52±0.61	2.41±0.68	2.28±0.71	0.459	2.42±0.66	2.4±0.76	2.58±0.78	0.665

* Significant at *p*≤0.05 (One-way ANOVA).

The rates of immediate debonding were 10.7% and 7.1% for the first and the second groups, respectively, with no statistically significant difference between them (*p* = 0.295). 

## DISCUSSION

Indirect bonding approach has recently gained more popularity, as it offers some substantial benefits, compared to direct bonding, including increased visibility during bracket bonding, better patient comfort, and decreased chair-time.^10^ Additionally, indirect bonding has become a more routine clinical procedure, as orthodontists are progressively shifting toward workflow digitization, including 3D virtual models, 3D photography, digital simulation of treatment results, and digital tooth movement monitoring using artificial intelligence.^18^ ^,^ ^19^

Whether the transfer trays are made using 3D-printed models^14^ ^,^ ^20^ or 3D-printing of transfer trays or jigs^17^ ^,^ ^21^, a meticulous balance is required regarding the gap between the brackets and the transfer tray. The tray has to exhibit the precise physical measurements of the brackets, with minor gap planned between them, permitting increased accuracy and bracket retention. However, some gap is needed to allow the tray to be removed without the risk of brackets debonding during its removal.^11^ ^,^ ^22^

The Carestream CS 3700 (Carestream Dental, Georgia, USA) intraoral scanner used in this study displayed excellent resolution and antireflection properties, making it suitable for accurate scanning of metal items, such as brackets, without the need of antireflection powder.^23^ Due to its thickness, this powder was verified to produce accuracy decrease, with increased total error throughout the scanning procedure.^24^ This scanner was demonstrated to achieve the highest accuracy, in comparison to twelve commercially available models of intraoral scanners, according to Mangano et al.^23^

Threshold values for linear and angular transfer accuracies in this study were 0.5 mm and 2°, respectively. This has taken into account the recommendations of the American Board of Orthodontics Cast-Radiograph Evaluation, which excludes points for every tooth presenting difference of 0.5 mm or more from the appropriate alignment.^25^ Moreover, a 2° crown-tip angular difference generates a marginal ridge inconsistency of 0.5 mm in a molar of standard size.^25^ Although the same values have been employed in many recent studies to evaluate bracket transfer accuracy during model superimposition^13^ ^,^ ^19^ ^,^ ^26^, Castilla et al.^27^ considered 0.25 mm as the critical linear bracket positioning deviation.

Linear transfer errors in this study were within the acceptable range for both groups. The mean linear errors ranged between 0.21 and 0.32 mm in the vacuum-formed tray group, and between 0.18 and 0.25 mm in the 3D-printed tray group. These outcomes were higher than those found by Kim et al.^28^, which varied between 0.05 and 0.19 mm using transfer jigs fabricated by CAD/CAM; by Castilla et al.^27^, which ranged between 0.06 and 0.49 mm when using double vacuum-formed transfer trays; and Schmid et al.^13^, which did not exceed 0.11 mm using double vacuum-formed transfer trays on 3D-printed models. The nature of this clinical study, with the correlated variables including patient movement, restricted mouth opening, and operator pressure, may be the reason for increased linear transfer errors, in comparison to most of these *in-vitro* studies. Both groups disclosed the most noticeable linear errors in the OG dimension. The smallest errors occurred in the MD and BL directions for both groups.

Concerning angular measurements, tip and rotation errors showed acceptable transfer errors. Only the torque deviations exceeded the tolerable thresholds in both groups. These results raise important questions regarding the influence of bonding resin thickness and its distribution on the bracket base on the torque error. Jungbauer et al.^26^ attributed the excessive torque errors related to hard transfer trays at crowded anterior teeth to the marked tension, giving rise to distortion of the lower portion of the hard tray.

There were statistically significant differences in the transfer errors between both groups for the occlusogingival linear deviation in both arches (*p* = 0.021 and *p* = 0.001 for the first and the second groups, respectively) and buccolingual linear deviation in the lower arch (*p* = 0.001), with the vacuum-formed tray group showing higher transfer errors than the 3D-printed tray group.

Both groups showed more than 93% frequency of linear clinically acceptable errors, within 0.5 mm. Frequency of clinically acceptable linear MD errors in the 3D-printed tray group was 100%. Rotation showed the highest frequency of angular clinically acceptable errors, within 2° (which did not exceed 91%), followed by the tip, with the torque showing the least frequency (less than 60%). There were no statistically significant frequency differences between both groups in all linear and angular dimensions. Chaudhary et al.^19^ reported incidence of more than 97% of linear clinically acceptable errors, within 0.5 mm, for the 3D-printed and polyvinyl siloxane (PVS) trays. Schmid et al.^13^ showed 100% prevalence in horizontal and transverse dimensions for both silicone and double vacuum-formed trays, with the smallest prevalence measured in torque for both groups.

The reduced angular control, compared to linear control, can be interpreted by the multiplanar bracket surfaces during the post-bonding scans, which, despite having slight impact regarding the linear errors, can substantially influence angular control, generating an imprecise coordinate system orientation, with the inclusion of considerable angular imprecisions.^17^ Additionally, the labial extension of the transfer tray immediately gingival to the brackets, without covering the cervical and the undercut areas, may have diminished the control of the bracket positioning in angular directions.^14^

Errors in both groups disclosed directional bias to the mesial, gingival and buccal directions. The gingival bias was 78.5% in the vacuum-formed tray group and 67% in the 3D-printed tray group. This gingival bias corroborates the outcomes of Grunheid et al.^10^ and Schmid et al.^13^, and can be attributed to the excessive vertical pressure on the transfer tray during seating, which can be exaggerated due to the sectional tray pattern. As softer tray designs are used, bracket positions do not remain stable when small deformations occur in the soft tray, resulting in further gingival displacements.^26^ Nevertheless, Niu et al.^14^ and Castilla et al.^27^ exhibited more occlusal directional bias of transferred brackets, and explained their results by inadequate tray seating due to a minor lack of alignment between the bracket and the tray, and by deficient vertical pressure on the tray due to the light-curing procedure. 

In the buccolingual orientation, 77.6% and 70% of the brackets were more buccally positioned in the first and the second groups, respectively. Analogous buccal directional bias was displayed by other studies^10^ ^,^ ^13^ ^,^ ^14^ ^,^ ^22^, and was mostly resulting from the thickness of the adhesive resin between the bracket base and the facial surfaces.

No statistically significant differences were identified in the transitional errors between the teeth groups (incisors, canines and premolars) in both arches for both groups, indicating that tooth type of trays did not influence the transfer accuracy. According to Kim et al.^28^, the alteration in teeth cusp height had no effect on the accuracy of bracket positioning with the CAD/CAM indirect bonding method, either in the linear or the angular directions. However, Schmid et al.^13^ revealed significant transfer error differences between incisors, canines and premolars in the horizontal and mesiodistal angulation directions for the silicone trays, with premolars displaying the greatest errors. With the double vacuum-formed trays, no significant differences were reported in both linear and angular dimensions.^13^

The rates of immediate debonding in this study were 10.7% and 7.1% for the first and the second groups, respectively, and can be explained by the extreme sensitivity of the indirect bonding approach. The failure rate described by Niu et al.^14^ was smaller than 11.3% for 3D-printed and vacuum-formed trays. The majority of the 12% failure rate demonstrated by Shcmid et al.^13^ resulted from the elimination of the spray used during the scanning procedure, with clinically relevant failures of about 1% for the double-vacuum and the silicone groups. Direct rebonding of these debonded brackets would eliminate the benefits of decreasing chair-time and improving accuracy related to indirect bonding.^14^

Transfer trays in both groups were segmented into three sections (two bilateral buccal sections, from canines to second premolars; and one anterior section enclosing the central and lateral incisors), to achieve a more simple and controllable bonding technique. Segmented transfer trays were believed to offer transfer accuracy comparable to one-piece trays, but with considerable decrease in bond failure rates.^29^

## CLINICAL IMPLICATIONS

The outcomes of this study suggest that the indirect bonding procedure allows for adequate overall accuracy of the desired bracket positions. The type of the tooth that is indirectly bonded has negligible impact on the accuracy. Indirect bracket bonding with 3D-printed trays is the technique of choice, compared to vaccum-formed trays, as it is generally more accurate. If other factors force the clinician to rely on vacuum-formed trays, it seems that there is no greater risk of increased immediate bracket debonding.

## LIMITATIONS

In this study, indirect bonding was executed by a single operator, with the purpose of reducing confounding variables. However, this could restrict the generalizability of the outcomes. A broad inclusion criterion was used, with no particular age group or malocclusion type, to imitate routine clinical practice. Nevertheless, this could have established variations regarding tray seating, adaptation and simplicity of removal, with possible effects on the final bracket positions. As the bonding agent was manually spread over the bracket bases, the clinician’s appraisal was the regulator that controlled the amount of the bonding material used. This could cause placement errors particularly in buccolingual linear dimension and torque. Pre-coated brackets could accomplish a practical solution for this dilemma.^30^

## CONCLUSIONS

3D-printed indirect bonding trays were more accurate than vacuum-formed trays, in terms of linear deviations. Both types of trays had similar angular control, frequency of clinically acceptable errors, and rates of immediate debonding. The null hypothesis of this study was rejected.
